# O-GlcNAc impacts mitophagy via the PINK1-dependent pathway

**DOI:** 10.3389/fnagi.2024.1387931

**Published:** 2024-08-08

**Authors:** Ibtihal M. Alghusen, Marisa S. Carman, Heather M. Wilkins, Taylor A. Strope, Caleb Gimore, Halyna Fedosyuk, Jad Shawa, Sophiya John Ephrame, Aspin R. Denson, Xiaowan Wang, Russell H. Swerdlow, Chad Slawson

**Affiliations:** ^1^School of Medicine, Department of Biochemistry and Molecular Biology, University of Kansas Medical Center, Kansas City, KS, United States; ^2^Department of Neurology, University of Kansas Medical Center, Kansas City, KS, United States; ^3^University of Kansas Alzheimer’s Disease Research Center, University of Kansas Medical Center, Kansas City, KS, United States

**Keywords:** O-GlcNAc, mitophagy, PTEN-induced kinase-1, Alzheimer’s disease, OGT

## Abstract

**Background:**

The accumulation of dysfunctional mitochondria is an early feature of Alzheimer’s disease (AD). The impaired turnover of damaged mitochondria increases reactive oxygen species production and lowers ATP generation, leading to cellular toxicity and neurodegeneration. Interestingly, AD exhibits a disruption in the global post-translational modification β-N-acetylglucosamine (O-GlcNAc). O-GlcNAc is a ubiquitous single sugar modification found in the nuclear, cytoplasmic, and mitochondrial proteins. Cells maintain a homeostatic level of O-GlcNAc by cycling the addition and removal of the sugar by O-GlcNAc transferase (OGT) or O-GlcNAcase (OGA), respectively.

**Methods:**

We used patient-derived induced pluripotent stem cells, a transgenic mouse model of AD, SH-SY5Y neuroblastoma cell lines to examine the effect of sustained O-GlcNAcase inhibition by Thiamet-G (TMG) or OGT deficiency on mitophagy using biochemical analyses.

**Results:**

Here, we established an essential role for O-GlcNAc in regulating mitophagy (mitochondria-selective autophagy). Stimulating mitophagy using urolithin A (UA) decreases cellular O-GlcNAc and elevates mitochondrial O-GlcNAc. Sustained elevation in O-GlcNAcylation via pharmacologically inhibiting OGA using Thiamet-G (TMG) increases the mitochondrial level of mitophagy protein PTEN-induced kinase 1 (PINK1) and autophagy-related protein light chain 3 (LC3). Moreover, we detected O-GlcNAc on PINK1 and TMG increases its O-GlcNAcylation level. Conversely, decreasing cellular O-GlcNAcylation by knocking down OGT decreases both PINK1 protein expression and LC3 protein expression. Mitochondria isolated from CAMKII-OGT-KO mice also had decreased PINK1 and LC3. Moreover, human brain organoids treated with TMG showed significant elevation in LC3 compared to control. However, TMG-treated AD organoids showed no changes in LC3 expression.

**Conclusion:**

Collectively, these data demonstrate that O-GlcNAc plays a crucial role in the activation and progression of mitophagy, and this activation is disrupted in AD.

## Introduction

Mitochondrial dysfunction is involved in the pathogenesis of diverse neurodegenerative diseases, including Alzheimer’s disease (AD) (Reddy et al., 2018). Abnormal mitochondrial function is detected prior to the clinical onset of AD contributing to the progression of the disease (Swerdlow, 2018). AD-related mitochondrial impairment involves decreasing ATP levels, oxidative phosphorylation (OXPHOS) activity, increasing ROS levels, and increasing tau phosphorylation (Ohta and Ohsawa, 2006; Swerdlow et al., 2014). The accumulation of damaged mitochondria is derived from the impairment of mitophagy, the selective removal of mitochondria by the autophagic machinery. Mitophagy is impaired in the hippocampus of AD patients, in induced pluripotent stem cell-derived human AD neurons, and in animal AD models (Cai and Jeong, 2020). Restoration and stimulation of mitophagy in neurons prevent dysfunctional mitochondria accumulation and toxicity. Pharmacologically inducing neuronal mitophagy through the activation of the PTEN-induced kinase-1 (PINK1) and Parkin-dependent pathway decreased the levels of toxic protein species associated with AD pathogenesis in human and mice neuronal cells (Fang et al., 2019; Kshirsagar et al., 2022). Post-translational modifications (PTMs) are critical throughout the different stages of mitophagy. PINK1-mediated phosphorylation of PARKIN recruits it to damaged mitochondria. Then, PARKIN ubiquitinates itself and many different mitochondrial outer-membrane proteins allowing cellular recognition of damaged mitochondria by the autophagy system. PTMs regulate the feed-forward amplification loop of mitophagy. Therefore, uncovering how PTMs regulate mitophagy is critical for our understanding of mitochondrial quality control. Thus, we asked whether and how O-GlcNAc post-translational modification regulates mitophagy in AD.

O-GlcNAc is a ubiquitous post-translational modification involving the attachment of a single N-acetylglucosamine moiety to serine or threonine residues on the nuclear, cytoplasmic, and mitochondrial proteins. The dynamic addition and removal of the sugar by O-GlcNAc transferase (OGT) or O-GlcNAcase (OGA), respectively, maintain a homeostatic level of cellular O-GlcNAc. Both OGT and OGA are found in the nucleus, cytosol, and mitochondria. OGT is essential for neuronal survival. Neuronal OGT knockout elicits embryonic loss and early death in postnatal mice (O'Donnell et al., 2004), while constitutive loss in forebrain neurons shows neurodegeneration in adult mice (Wang et al., 2016). At functional synapses in the mature brain, OGA and OGT are significantly active, leading to extensive O-GlcNAc modification of proteins in nerve terminals, and many neuronal and synaptic proteins are modified by O-GlcNAc (Cole and Hart, 2001). Importantly, synaptic transmission is a high energy-demanding process, and mitochondria are enriched at synapses providing ATP (Sheng and Cai, 2012), and many mitochondrial proteins undergo dynamic O-GlcNAcylation to control energy production (Dontaine et al., 2022).

Previously, we showed that O-GlcNAc regulates ROS production and the energetic function of the mitochondria (Flax et al., 2020). Pharmacologic inhibition of OGA lowers the activity of the electron transport chain while decreasing ATP and ROS production (Tan et al., 2017). Any manipulation of O-GlcNAc levels causes alterations in mitochondrial morphology (Burman et al., 2017). Overexpressing OGT or OGA leads to changes in mitochondrial shape compared to GFP-expressing controls (Tan et al., 2014). Preserving optimal mitochondrial function requires recycling damaged mitochondria via autophagy. Pharmacological inhibition of OGA stimulates autophagy in two AD models, the JNPL3 tauopathy mouse model and the 3 × Tg-AD mouse model, preventing the accumulation of AD-related toxic species such as Tau (Zhu et al., 2018). O-GlcNAc regulates autophagy by regulating the expression of its upstream targets. Recently, we showed that the elevation of O-GlcNAc increases activating transcription factor 4 (ATF4) activity, a master transcriptional regulator for essential mitophagosome formation-related proteins including LC3 (Alghusen et al., 2023). Interestingly, OGT-deficient hematopoietic and progenitor cells (HSPCs) show accumulation of defective mitochondria due to impaired mitophagy with a decrease in PINK1, revealing that O-GlcNAc regulates mitochondrial stress response ensuring mitochondrial quality (Murakami et al., 2021). Collectively, these studies suggest that O-GlcNAc is essential for mitophagy, and any manipulation of O-GlcNAc could positively or negatively impact mitophagy. In our current study, we show that OGA inhibition increases mitophagy in SH-SY5Y neuroblastoma cells, C57BL6J mice brains, and organoids differentiated from normal individuals. Both SH-SY5Y OGT-KD and OGT-KO mice brains decrease PINK1 and LC3 protein levels. These data suggest that O-GlcNAc elevation increases mitophagy, while the loss of OGT decreases it. However, elevating O-GlcNAc via OGA inhibition has no impact on organoids differentiated from AD patients.

## Materials and methods

### Cell culture

SH-SY5Y cells were cultured in low glucose Dulbecco’s modified Eagle’s Medium (DMEM) prepared as follows: 44 mm sodium bicarbonate (Sigma), DMEM (Sigma D5030-10 L), 4 mM glucose, and 15 mg/L phenol red (Sigma) and supplemented with 1% GlutaMAX (Gibco), 10% fetal bovine serum (FBS; Gemini), and 1% penicillin/streptomycin (Sigma). Cells were treated with 10 μm Thiamet-G (SD Specialty Chemicals) (TMG, from 20 mM stock, Tris-buffered saline pH 7.4) for a minimum of 3 weeks prior to experiments. The culture medium was changed daily to prevent glucose starvation. The cells were harvested for total lysate or mitochondria isolation at different time points 1, 2, 4, 6, and 8 h after stimulating mitophagy using [50 μm urolithin A (UA), Sigma# 1143-70-0]. Lysosomes were inhibited using 20 μm chloroquine (MCE # HY-17589) for 4 h.

### Lentivirus preparation

The OGT knockdown (KD) was generated by shRNA-mediated lentiviral KD (ThermoFisher). Plasmids of OGT-KD shRNAs and scramble GFP shRNA, along with plasmids that encode for lentiviral particles, were purified using a Maxiprep Kit (NA0310—Sigma-Aldrich). HEK293T cells were plated at a density of 5 × (10^6^) cells in a 10-cm dish having a total volume of 10 mL DMEM (25 mM glucose) during day 1. During day 2, HEK293T cell plates were transfected with OGT shRNA plasmids along with PCMV and PMD2G plasmids encoding the viral coat using TransIT-X2® Dynamic Delivery System (Mirus MIR 6005) in 1.5 mL of opti-MEM serum-free media (ThermoFisher Catalog # 11058021). The media containing lentivirus were collected the next day and stored at −80°C. The process was repeated for two additional days. Finally, conditioned media were centrifuged at 1,000 *g* for 3 min and the supernatant was passed through a 0.45-μm filter making lentivirus infection media. The filtered media containing the virus were then used to infect SH-SY5Y, creating the OGT-KD as described below.

### shRNA lentivirus infection

The SH-SY5Y cells were plated in a 10-cm dish using DMEM (25 mM glucose). After reaching 95% confluency, the culture medium was discarded and replaced with 4 mL of fresh medium and 4 mL of lentivirus collected after the lentivirus preparation described above for the infection. The culture medium in each plate was discarded the next day and replaced with a 10 mL of fresh DMEM (25 mM glucose) medium supplied with puromycin at 1 μg/mL as a selection. The process was continued for the next 4–9 days.

### Animal protocols and models

The University of Kansas Medical Center Animal Care and Use Committee approved all experiments in this study. Two-month-old male C57Bl/6 J and 5XFAD mice were purchased from the Jackson Laboratory (Bar Harbor, ME). All mice were housed using a standard 12-h light/dark cycle with access to chow and water *ad libitum*. At least, three male C57Bl/6 J and 5XFAD mice were treated with 50 mg/kg Thiamet-G or saline intraparietal injection every other day for 30 days (Alghusen et al., 2023). After completion of the dosing, mice were fasted for 16 h before undergoing isoflurane (Fisher) anesthesia-assisted cervical dislocation.

*Floxed-*OGT mice were obtained from Dr. John Hanover at the NIH. CAMK2-Cre/ERT2 mice were purchased from the Jackson Laboratory (Bar Harbor, ME). *Floxed-*OGT are bred with CAMK2-Cre/ERT2 mice to generate tamoxifen-inducible OGT-KO. Tamoxifen was dissolved in corn oil (20 mg/mL). The mice were injected with 200 μL tamoxifen solution (4 mg) once a day over 5 days. Three months after completion of the dosing, the mice were fasted for 16 h before isoflurane (Fisher) anesthesia-assisted cervical dislocation.

### Cell lysis and immunoblotting

The cells were lysed on ice in NP-40 lysis buffer containing 20 mM Tris, pH 7.4, 150 mM NaCl, 40 mM GlcNAc, 2 mM EDTA, 1 mM DTT, 1% Nonidet P-40 with phosphatase inhibitors 1 mM β-glycerophosphate, 1 mM sodium fluoride (NaF), and protease inhibitors 2 mM phenylmethylsulfonyl fluoride (PMSF) and 1 × inhibitor mixture composed of 1 μg/mL leupeptin, 1 μg/mL antipain, 10 μg/mL benzamidine, and 0.1% aprotinin added before lysis. Animal tissues were lysed with RIPA buffer containing 10 mM Tris, pH 7.6, 150 mM NaCl, 40 mM GlcNAc, 2 mM EDTA, 1 mM DTT, 1% Nonidet P-40, 0.1% SDS, 0.5% deoxycholic acid with phosphatase inhibitors 1 mM β-glycerophosphate, 1 mM sodium fluoride (NaF), and protease inhibitors 2 mM phenylmethylsulfonyl fluoride (PMSF) and 1 × inhibitor mixture composed of 1 μg/mL leupeptin, 1 μg/mL antipain, 10 μg/mL benzamidine, and 0.1% aprotinin added immediately before lysis. All the above ingredients were purchased from Sigma. Lysates were kept on ice for 20 min and vortexed every 5 min. The protein concentration of the lysate was determined using Bradford assay (Bio-Rad Catalog) or BCA assay (ThermoFisher).

### SDS-PAGE and western blotting

Lysates were then denatured by the addition of a 4 × protein solubility mixture (100 mM Tris, pH 6.8, 10 mM EDTA, 8% SDS, 50% sucrose, 5% β-mercaptoethanol, and 0.08% pyronin Y) and boiling for 2 min. Equal protein concentrations of lysates were loaded onto 4–15% Criterion Precast TGX gels (Bio-Rad). Electrophoresis occurred at 130 V for approximately 60 min, and then the gel proteins were transferred to polyvinylidene difluoride (PVDF) membranes at 0.4 A. Membranes were blocked with 3% BSA and 0.01% sodium azide in TBST (25 mM Tris, pH 7.6, 150 mM NaCl, and 0.05% Tween-20) for at least 15 min. Blots were incubated overnight at 4°C with primary antibody to the protein of interest at 1:2,000 dilution. The next day, the blots were washed three times in TBST for 10 min each. HRP-conjugated secondary antibody (Bio-Rad) at 1:10,000 dilution was added for 1 h at a rotating setting at room temperature, followed by washing three times with TBST for 10 min each. The blots were then developed using the chemiluminescence HRP antibody detection method (ThermoFisher Catalog # 34095). The blots were stripped with 200 mM glycine, pH 2.5, for 1 h at room temperature, blocked for 15 min with 3% BSA, and re-probed overnight at 4°C with primary antibody. ImageJ 3.2 (National Institutes of Health) or image Lab (Bio-Rad) were used to quantify the density of protein bands normalized with an internal standard protein band such as GAPDH, α-Tubulin, and TOM20. Statistical significance was measured using unpaired-*t*-test analysis and the *p* value is indicated on the plots. ^*^Is added for significant *p* values (*p* < 0.05).

### Antibodies

Primary antibodies and secondary antibodies for immunoblotting were used at 1:2,000 and 1:10,000 dilutions, respectively. OGT (AL-34) and OGA (345) were gracious gifts from the laboratory of Gerald Hart in the Department of Biochemistry and Molecular Biology at the University of Georgia. Anti-*O*-linked *N*-acetylglucosamine antibody (RL2, ThermoFisher #MA1-072), anti-GAPDH antibody (ab9484), PINK1 (cell signaling #6946S), LC3 (cell signaling #2775S), TOM20 (Santa Cruz (F-10): sc-17764), α-tubulin (sigma Catalog #T5168), GFAP (ab7260), MAP2 (ab32454), NeuN (ab177487), B3 tubulin (ab18207), ChAT (ab181023), doublecortin (ab207175), Pax-6 (ab195045), and synaptophysin (ab32127). Anti-chicken IgY-HRP (A9046) was purchased from Sigma. Goat anti-rabbit IgG-HRP (170-6515) and goat anti-mouse IgG-HRP (170-6516) were purchased from Bio-Rad.

### Mitochondrial purification from cells and brain

Mitochondria were isolated from SH-SY5Y cells using the cavitation method as described previously (Alghusen et al., 2023). Briefly, 2 × 10^8^ cells were trypsin digested off the plate using 1 mL of trypsin and washed two times with 10 mL of pre-chilled PBS. Centrifugation was performed at 1,000 × *g* for 3 min. The pellet was resuspended into 3 mL of the mitochondrial isolation buffer (MIB; 225 mM mannitol, 75 mM sucrose, 6 mM K2HPO4, 1 mM EGTA, 0.1% fatty acid-free BSA, pH 7.2). The cell suspensions were transferred into a pre-chilled cavitation chamber (nitrogen bomb; Parr Instrument Co., Moline, IL, United States) and subjected to 900 p.s.i. for 15 min. Subsequently, the pressurized cell suspension was collected from the cavitation chamber, followed by centrifugation at 1000 × *g* for 10 min to pellet the cell debris. The clear supernatant was collected and centrifuged at 12,000 × *g* for 15 min. The crude mitochondrial pellet was washed three times with 500 μL of pre-chilled isolation medium. The washed pellet was lysed with Nonidet P-40 lysis buffer.

Male mouse brain mitochondria were isolated from the whole brain using the Percoll gradient and ultracentrifugation as described previously (Alghusen et al., 2023). All reagents are pre-chilled on ice. The brain was rinsed two times with ice-cold PBS, and then cut and homogenized (Teflon glass homogenizer) in 5 mL of mitochondria isolation buffer (MIB; 225 mM mannitol, 75 mM sucrose, 6 mM K2HPO4, 1 mM EGTA, 0.1% fatty acid-free BSA, and pH 7.2). The resulting homogenate was centrifuged at 1,500 × *g* for 5 min at 4°C; 5 mL of supernatant was added to the top of the layered Percoll gradients prepared as follows: 15, 23, and 40% Percoll gradients were made using 100% Percoll in MIB. 2.3 mL of the 40% Percoll gradient was layered on the bottom of a centrifuge tube, followed by 2.3 mL of 23% Percoll (middle), then 2.3 mL of 15% Percoll (top). Ultracentrifugation was performed using an SW28.1 Beckman rotor; tubes were centrifuged at 7,800 rpm for 15 min at 4°C. The mitochondrial layer (the white layer at the bottom of 23% Percoll gradient) was collected and washed with 8 mL of MIB and centrifuged at 8,000 × *g* for 10 min. Mitochondria were then washed a second time with 8 mL of sterile phosphate-buffered saline (PBS) and re-centrifuged at 8,000 × *g* for 10 min. Precipitated mitochondria were lysed using 500 μL of Nonidet P-40 lysis buffer. Protein determination assays and Western blotting were performed.

### iPSC source and reprogramming

As shown previously (Alghusen et al., 2023), iPSCs were reprogrammed from dura fibroblasts obtained at the University of Kansas Alzheimer’s Disease Research Center (KU ADRC) or purchased from WiCell. KU ADRC fibroblast donors were members of the Clinical Cohort, who consented to donation upon death, and approval from an ethical standards committee to conduct this study was received. The studies involving human participants were reviewed and approved by the University of Kansas Medical Center Institutional Review Board. Banked tissue is de-identified by the KUADRC Neuropathology Core to eliminate identifying information. Reprogramming was completed using the Sendai Virus, CytoTune-iPS 2.0 *Sendai Reprogramming* Kit from ThermoFisher. iPSC were age-, sex-, and diagnosis-matched (Supplementary Table 1). For iPSCs derived from the KU ADRC cohort, ND or AD was diagnosed at autopsy neuropathological examination as outlined in the NACC Neuropathology Coding Guidebook ed2020. Cerebral organoids were made using iPSCs and Stemdiff Cerebral Organoid kits from STEMCELL. The iPSCs were briefly placed into single-cell suspensions with ROCKi in embryoid body formation plates. Embryoid bodies expanded for 7 days and were then embedded into Matrigel droplets. The organoids matured for 90 days. The Tissue Clearing Kit, hydrophobic (ab243298), was used for tissue clearing and immunostaining.

### Mitochondrial staining

These assays were completed using Corning 96-well plates. MitoTracker Red was used at a concentration of 50 nM. Hoechst was added to a final concentration of 10 μg/mL. The cells were incubated with dyes for 30 min and washed two times with Hank’s Balanced Buffer Solution (HBSS with Ca2+ and Mg2+). Images were collected and analyzed using a Cytation 1 Cell Imaging MultiMode Reader from BioTek. MitoTracker intensity was normalized to the total cell number in each image.

## Results

**Figure 1:**
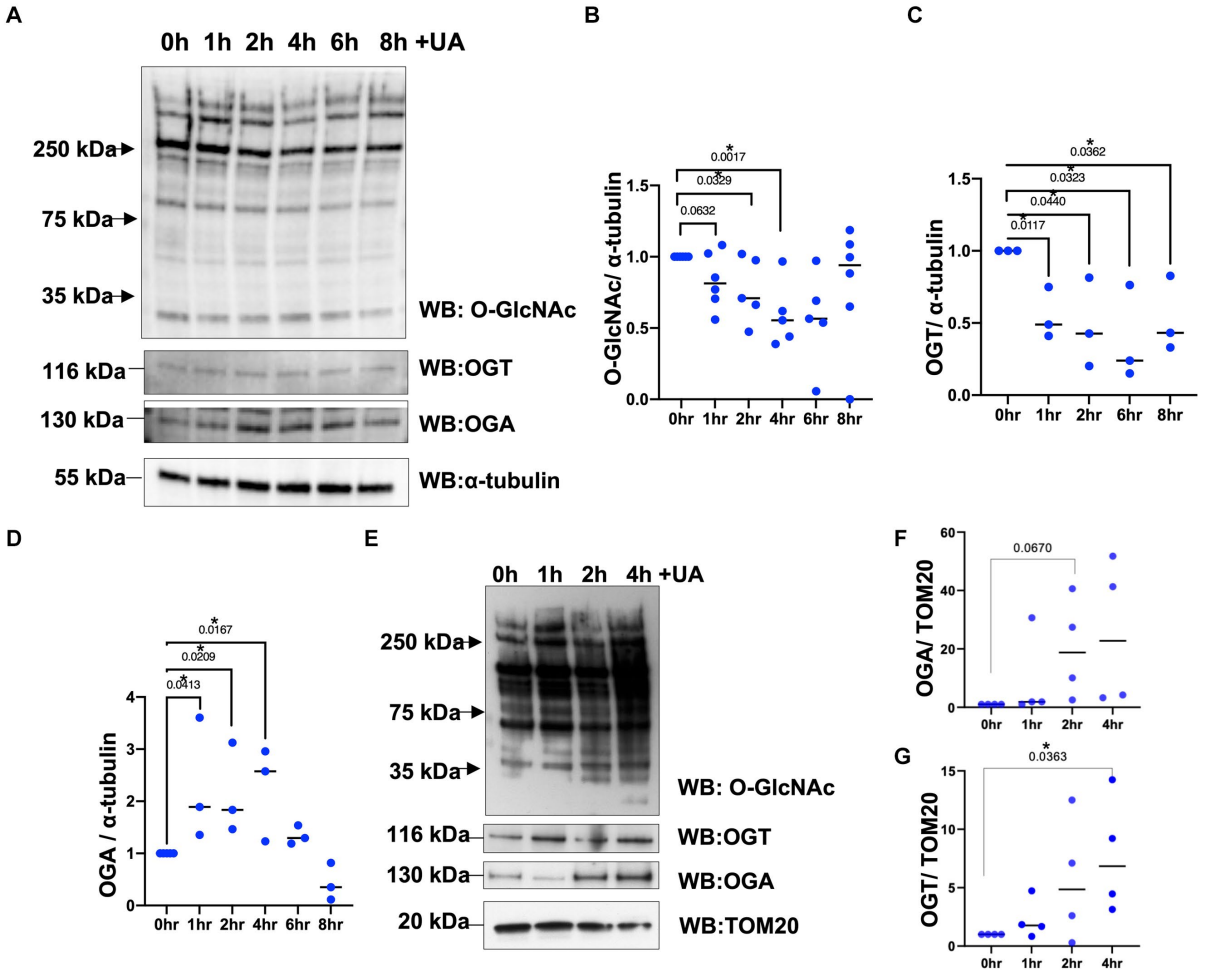
Mitochondrial stress alters cellular and mitochondrial O-GlcNAcylation, OGA, and OGT upon urolithin A addition. **(A)** Representative Western blot analysis of total lysates samples harvested after stimulating mitochondrial stress in different time points, 0, 1, 2, 4, 6, and 8 h, using 50 μm of urolithin A (UA) in SH-SY5Y neuroblastoma. The blots were probed for panel O-GlcNAc, OGT, OGA, and α-tubulin. **(B–D)** Densitometry plot of O-GlcNAc, OGT, and OGA normalized to loading control, α-tubulin. **(E)** Representative Western blot analysis of mitochondrial isolated samples harvested after stimulating mitochondrial stress using UA time course in SH-SY5Y neuroblastoma cells. The blots were probed for panel O-GlcNAc, OGT, OGA, and TOM20. **(F,G)** Densitometry plot of OGA and OGT normalized to the mitochondrial loading control, TOM20. (*n* > 3) where dots represent the number of experimental trials (*n*). Statistical significance was measured using unpaired *t*-test analysis, and the *p* value is indicated on the plots. ^*^Is added for significant *p* values (*p* < 0.05).

O-GlcNAc levels increase in response to diverse cellular stress responses like heat shock (Zachara et al., 2011), hypoxia (Ngoh et al., 2009), and oxidative stress (Lee et al., 2016). However, it is unknown whether O-GlcNAc levels are altered in response to mitochondrial stress. To determine whether mitochondrial stress affects cellular O-GlcNAcylation, we subjected the neuroblastoma cell line (SH-SY5Y) with urolithin A (UA, 50 μm) to initiate mitochondrial stress and promote mitophagy (Fang et al., 2019). The cells were harvested at different time points 1, 2, 4, and 8 h after the addition of UA. The O-GlcNAc levels gradually decrease over time, reaching a maximum decrease at 4–6 h. At 8 h of UA treatment, the O-GlcNAc levels returned to their basal state (Figures 1A,B). Then, to determine whether mitochondrial stress influences the protein expression of enzymes regulating O-GlcNAc, we measured the levels of OGT and OGA after the addition of UA. OGT protein levels decrease significantly, while OGA levels increase upon stress (Figures 1A–D). Then, we questioned how mitochondria stress affects mitochondrial O-GlcNAcylation levels. We purified mitochondria at different time points after UA addition. Within 1–2 h, mitochondrial O-GlcNAcylation was elevated and returned to basal level at 4 h (Figure 1E). Then, we asked whether mitochondrial stress impacts mitochondrial OGT/OGA levels. A significant mitochondrial elevation of OGA and OGT compared to control occurred (Figures 1E–G). These data demonstrate that O-GlcNAcylation changes in response to mitochondrial stress, and OGT/OGA levels are elevated in mitochondria during mitophagy.

**Figure 2:**
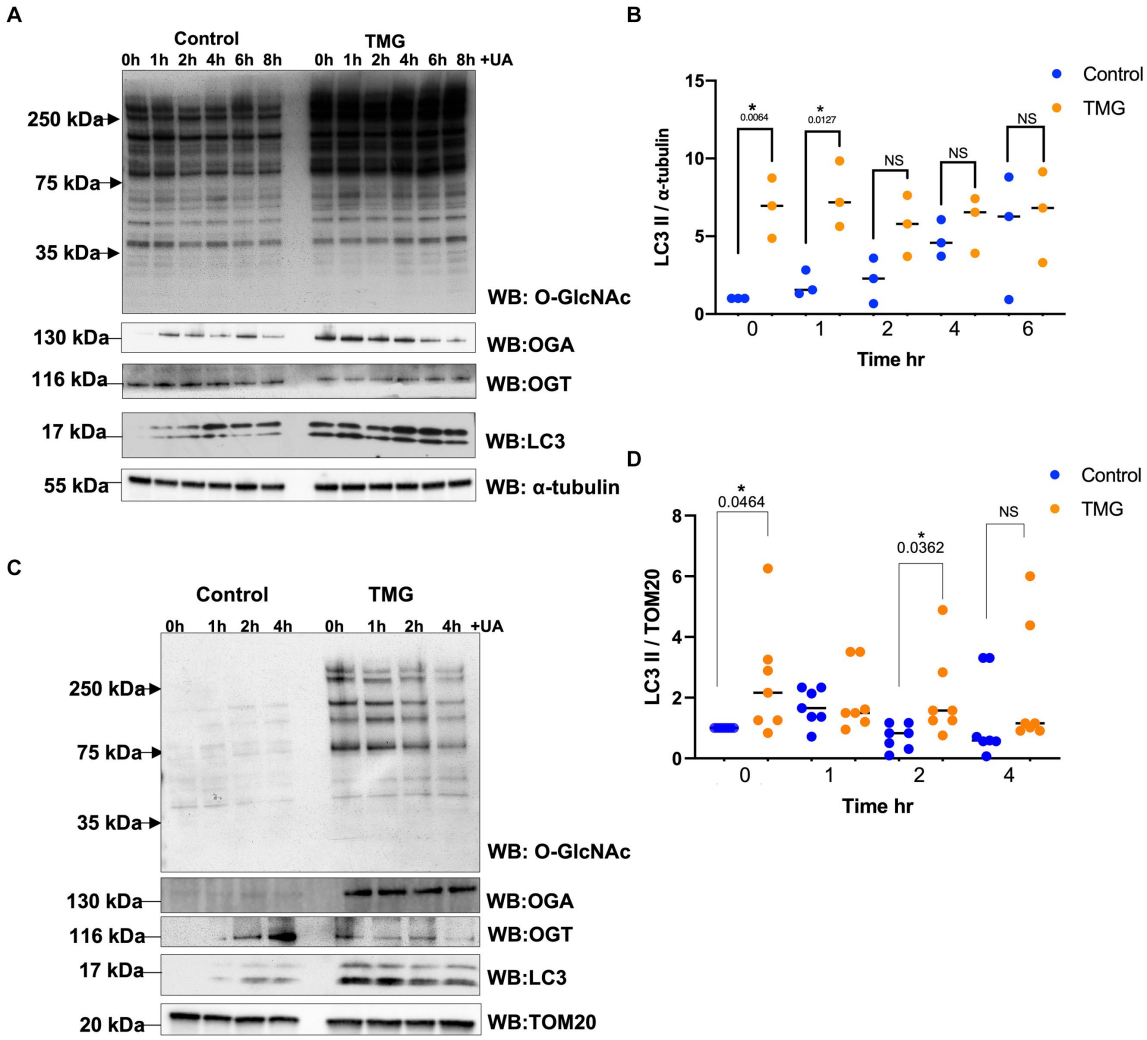
TMG initiates mitophagy in human cell lines. **(A)** Representative Western blot analysis of total lysates samples harvested after stimulating mitochondrial stress using UA time course, 0, 1, 2, 4, 6, and 8 h, in SH-SY5Y neuroblastoma cells subjected to long-term OGA inhibition with TMG. The blots were probed for panel O-GlcNAc, OGT, OGA, LC3, and α-tubulin. **(B)** Densitometry plot of LC3II normalized to loading control, α-tubulin. **(C)** Western blot analysis of mitochondrial isolated samples harvested after stimulating mitochondrial stress using UA time course, 0, 1, 2, and 4 h, in SH-SY5Y neuroblastoma cells subjected to long-term OGA inhibition with TMG. The blots were probed for panel O-GlcNAc, OGT, OGA, LC3, and TOM20. **(D)** Densitometry plot of LC3II normalized to TOM20. Experiments were performed with at least three biological replicates. Statistical significance was measured using unpaired *t*-test analysis, and the *p* value is indicated on the plots. ^*^Is added for *p* values that are significant (*p* < 0.05).

Then, we questioned whether O-GlcNAc manipulation can affect mitophagy-related proteins. We treated SH-SY5Y long-term with TMG (10 μm) for at least 3 weeks prior to harvesting. A sustained elevation in O-GlcNAc in long-term TMG treatment allows cellular adaptation to a high O-GlcNAc cycling rate. We also treated with UA to initiate mitophagy and harvested cells at time points 1, 2, 4, 6, and 6 h. TMG increases O-GlcNAc as expected (Figure 2A). TMG increases OGA expression, while a decline in OGT expression was evident in the total lysate of TMG-treated cells (Figure 2A). This is because the elevation in O-GlcNAc signals through a transcriptional network to induce the expression of OGA. LC3II, the active form generated by the conjugation of cytosolic LC3I to phosphatidylethanolamine on the surface of autophagosomes, shows a significant elevation, 7-fold higher in TMG-treated cells compared to control. In control cells, UA elevates LC3II to the same level of TMG at 6 h, while UA has no impact on TMG-treated cells (Figures 2A,B). To assess mitophagy, we isolated mitochondrial fraction from SH-SY5Y-treated cells with TMG and stimulated for mitophagy using UA at different time points 0, 1, 2, and 4 h. LC3II is higher in TMG-treated cells than control. However, the addition of UA to TMG-treated cells did not elevate LC3II compared to 0 h (Figures 2C,D). These data indicate that TMG elevates the expression of mitophagy-related proteins as shown in SH-SY5Y total lysate and mitochondrial fraction compared to control.

**Figure 3:**
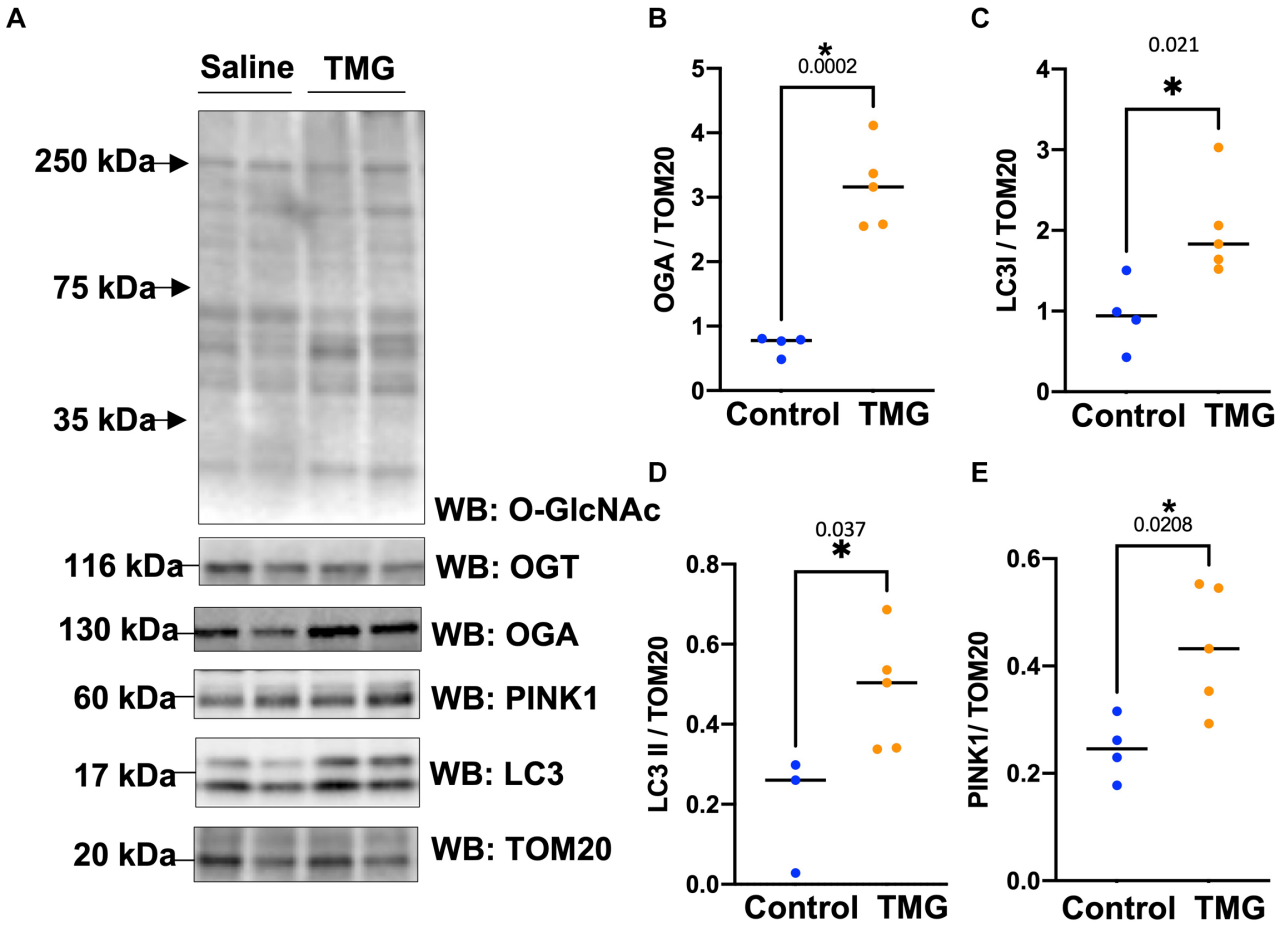
TMG increases mitochondrial PINK1 and LC3 in mice isolated brains. Representative Western blot analysis of brains’ mitochondria of WT-C57BL/6 J male mice subjected to intraperitoneal TMG injection for 1 month **(A)**. The blots were probed for panel O-GlcNAc, OGT, OGA, TOM20, PINK1, and LC3. **(B–E)** Densitometry plot of OGA, OGT, LC3I, LCII, and PINK1 normalized to TOM20. The experiments were performed with at least three biological replicates. Statistical significance was measured using unpaired *t*-test analysis, and the *p* value is indicated on the plots. ^*^Is added for *p* values that are significant (*p* < 0.05).

**Figure 4:**
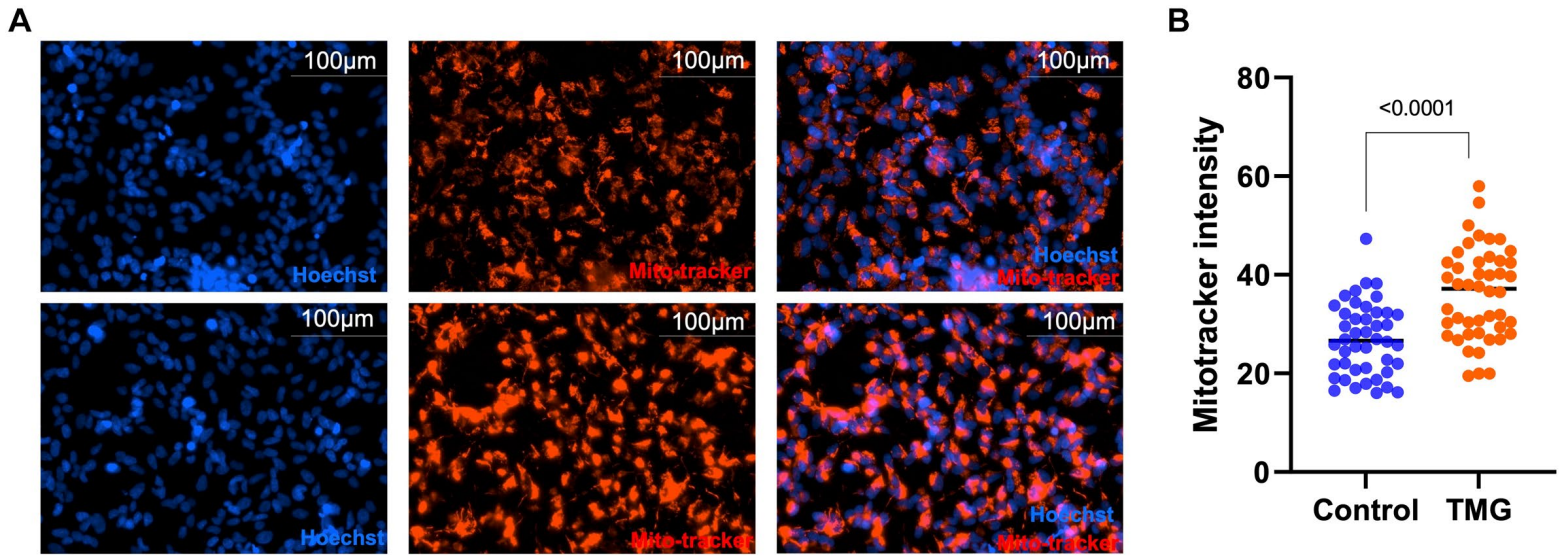
TMG increases mitochondrial intensity. Representative images for MitoTracker and Hoechst-stained SH-SY5Y cells subjected to long-term TMG **(A)**. **(B)** Densitometry plot of MitoTracker intensity normalized to total cell number (Hoechst). Statistical significance was measured using unpaired *t*-test analysis, and the *p* value is indicated on the plots. ^*^Is added for *p* values that are significant (*p* < 0.05).

**Figure 5:**
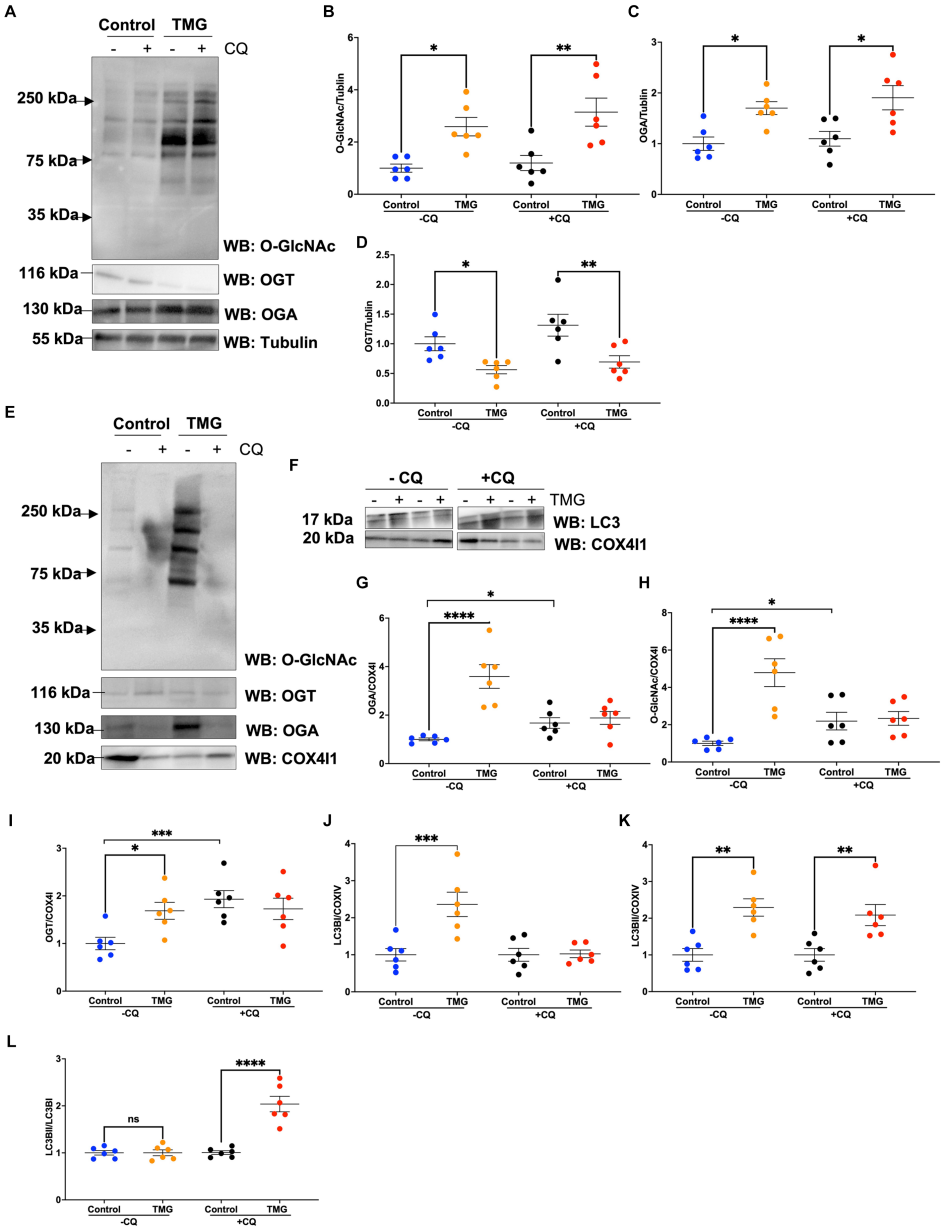
TMG elevates the LC3II/LC3I ratio of mitochondrial fraction upon blocking lysosome function. Representative Western blot analysis of total lysates samples harvested after inhibiting lysosomes using chloroquine for 4 h in SH-SY5Y cells subjected to long-term TMG **(A)**. The blots were probed for panel O-GlcNAc, OGT, OGA, and α-tubulin. Densitometry plot of O-GlcNAc, OGA, OGT, LC3I, and LCII normalized to α-tubulin **(B–D)**. **(E,F)** Representative Western blot analysis of isolated mitochondria after inhibiting lysosomes using chloroquine for 4 h in SH-SY5Y cells subjected to long-term TMG. The blots were probed for panel O-GlcNAc, OGT, OGA, tubulin, LC3, and COX4I1. Densitometry plot of O-GlcNAc, OGA, OGT, LC3I, and LCII normalized to COX4I1 **(G–K)**. **(L)** Densitometry plot of LCII normalized to LC3I. Statistical significance was measured using unpaired *t*-test analysis, and the *p* value is indicated on the plots. ^*^Is added for *p* values that are significant (*p* < 0.05).

Then, we asked whether TMG treatment elevates mitophagy markers in mitochondria isolated from mouse brains. We injected C57BL/6 J mice intraperitoneally with TMG or saline, and we isolated mitochondria from the brains of these mice. Mitochondria isolated from TMG-treated mice show an elevation of O-GlcNAc as expected (Figure 3A). Mitochondrial OGA increased to 3-fold higher, and OGT also had a significant increase. PTEN-induced kinase-1 (PINK1) and both forms of LC3 are significantly elevated compared to mitochondria isolated from control animals (Figures 3A–E). These data suggest that TMG enhances the translocation of PINK1 and LC3 to mitochondria and TMG potentially induces mitophagy. However, mitochondrial dynamics include mitochondrial genesis, mitofusion, mitofission, and mitophagy; thus, to address mitochondrial dynamics, we performed a series of experiments. First, we used MitoTracker to examine whether TMG affects mitochondrial dynamics. TMG significantly elevates mitochondrial intensity in SH-SY5Y compared to control, suggesting more or longer mitochondria (Figures 4A,B). We tested mtDNA content, but there was no change in mitochondria amount with TMG. Our data replicate previous experiments showing longer mitochondria with TMG treatment (Tan et al., 2014). Then, to further identify the role of O-GlcNAc in mitophagy, we blocked lysosomes using chloroquine (CQ) and assessed O-GlcNAc alterations and mitophagy markers in SH-SY5Y total cellular lysates and mitochondrial fraction (Figures 5A–K). CQ has no effect on global cellular O-GlcNAc and OGA, while OGT is slightly elevated (Figures 5A–D). However, blocking lysosomes using CQ slightly elevates O-GlcNAc, OGA, and OGT in isolated mitochondria (Figures 5E–K). Blocking lysosomal function stalls the final stage of mitophagy, fusion with the lysosome, initiating mitochondrial stress and elevating mitochondrial OGT, OGA, and O-GlcNAc. Importantly, LC3II is significantly elevated in mitochondria isolated from TMG- and CQ-treated cells, suggesting OGA inhibition induces mitophagy (Figures 5K,L).

**Figure 6:**
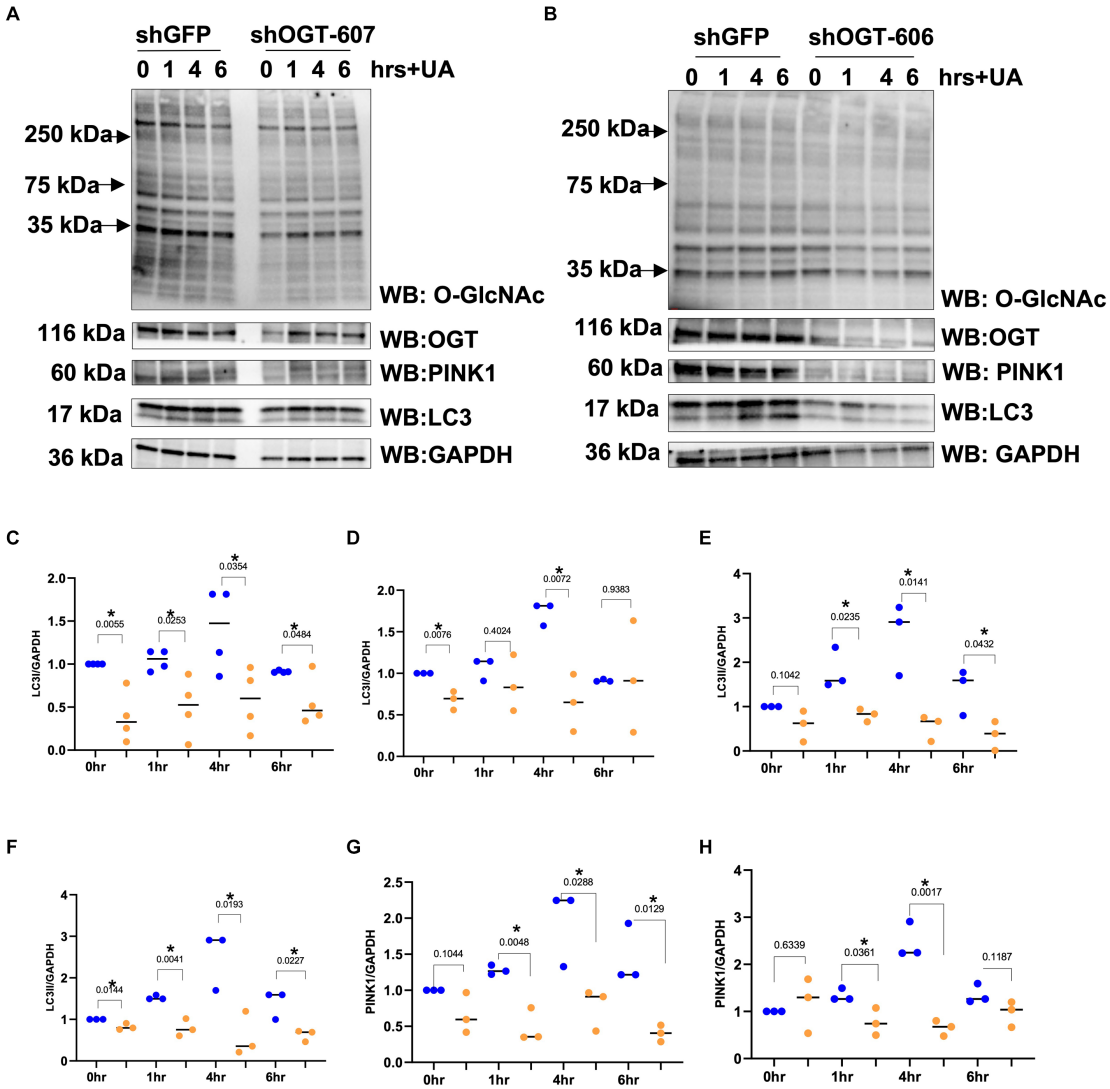
OGT-KD decreases LC3 and PINK1. Western blot analysis of samples harvested after stimulating mitochondrial stress time course, 0, 1, 4, and 6 h, in SH-SY5Y OGT-KD cells 606 and 607 **(A,B)**. **(C,E,G)** Densitometry plot of LC3I, LC3II, and PINK1 normalized to loading control, GAPDH in SH-SY5Y shOGT-606 and SH-SY5Y shGFP. **(D,F,H)** Densitometry plot of LC3I, LC3II, and PINK1 normalized to loading control and GAPDH in SH-SY5Y shOGT-607 and SH-SY5Y shGFP. Experiments were performed with at least three biological replicates, where the dots represent the number of experimental trials (*n*). Statistical significance was measured using paired *t*-test analysis, and the *p* value is indicated on the plots. ^*^Is added for *p* values that are significant (*p* < 0.05).

**Figure 7:**
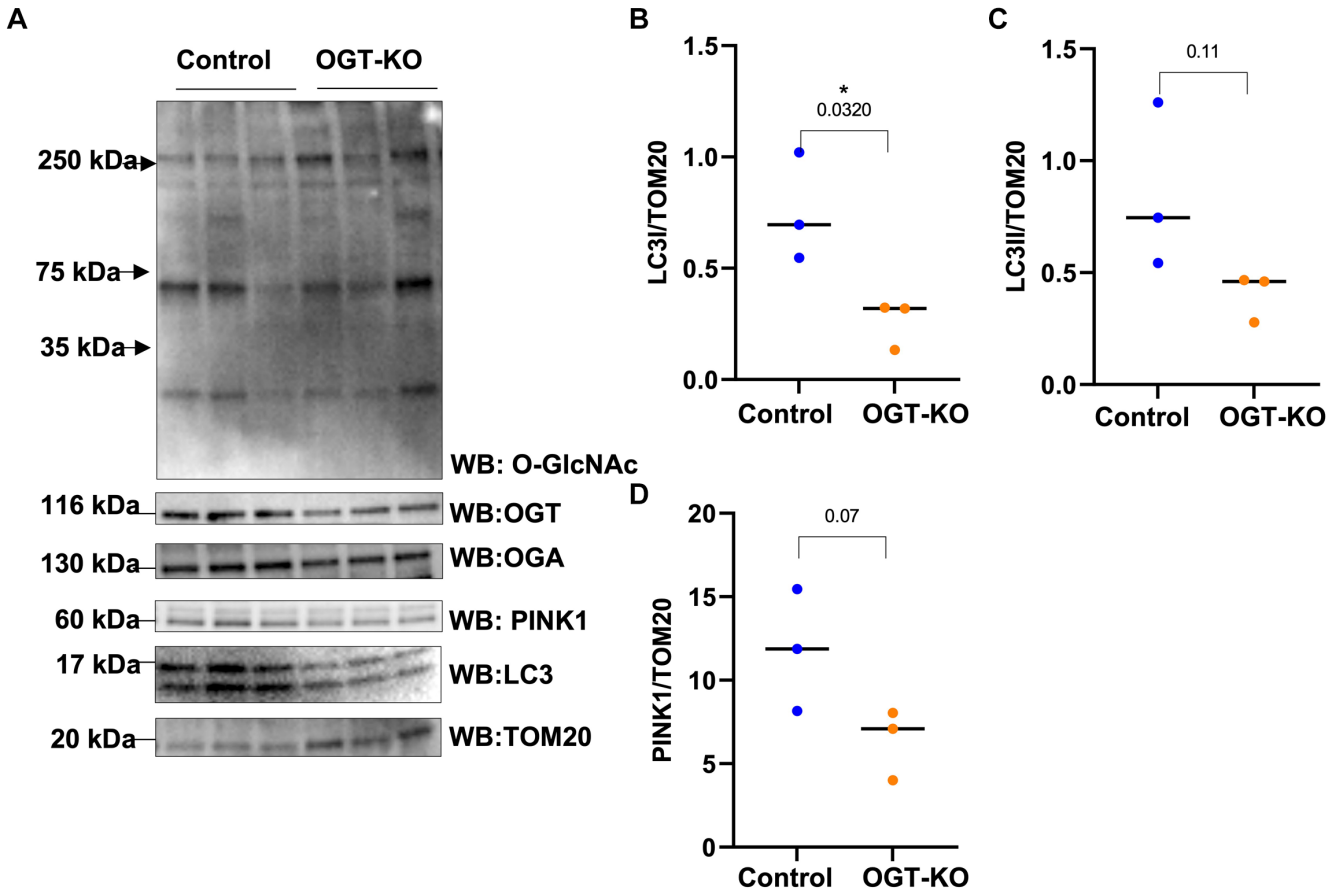
OGT-KO decreases LC3 and PINK1 in mice brain mitochondria. Western blot analysis of mitochondria of brains of WT-C57BL/6 J or OGT-KO C57BL/6 J male mice subjected to inducible KO for 1 month **(A)**. The blots were probed for panel O-GlcNAc, OGT, OGA, TOM20, PINK1, and LC3. **(B–D)** Densitometry plot of LC3I, LCII, and PINK1 normalized to TOM20. OGT-KO (*n* = 3) and WT-C57BL/6 J (*n* = 3) where the dots represent the number of experimental trials (*n*). Statistical significance was measured using unpaired *t*-test analysis, and the *p* value is indicated on the plots. ^*^Is added for *p* values that are significant (*p* < 0.05).

**Figure 8:**
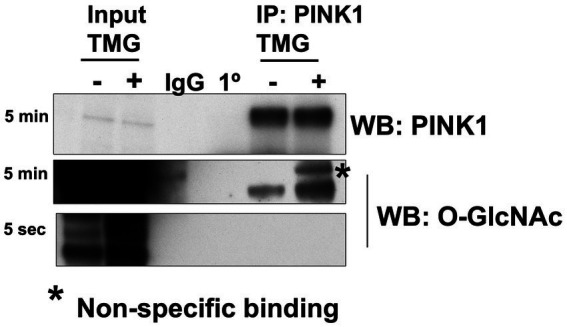
PINK1 is modified by O-GlcNAc. PINK1 immunoprecipitation was performed using TMG-treated or non-TMG-treated SH-SY5Y. A rabbit non-specific antibody control (IgG) and a PINK1 antibody control (1°) are included. PINK1 immunoprecipitation results showed increased PINK1 O-GlcNAcylation upon TMG in SH-SY5Y (*n* > 3).

Then, we explored how a reduction in OGT affects mitophagy markers and we assessed PINK1 and LC3 protein expression in OGT-KD SH-SY5Y cells. We observed a similar mitochondrial stress time course in SH-SY5Y OGT-KD cell lines 1 and 2 (two different OGT-KD short-hairpin shRNA-expressing cell lines). OGT-KD caused O-GlcNAc and OGT to decrease (Figures 6A,B). Interestingly, OGT-KD decreased LC3 and PINK1 protein expression significantly compared to control (Figures 6A–H). Then, we questioned whether the loss of OGT affected the expression of LC3 and PINK1 in mice brain mitochondria similarly to the cell line data. We isolated mitochondria from the brains of CAMKII-CRE-inducible OGT-KO mice. Mitochondria purified from OGT-KO mice decreased PINK1 and LC3 compared to control mice (Figure 7). To examine whether PINK1 is modified by O-GlcNAc, we performed PINK1 immunoprecipitation from long-term TMG-treated SH-SY5Y total cell lysates and compared it to non-TMG-treated. TMG treatments increased PINK1 O-GlcNAcylation (Figure 8).

**Figure 9:**
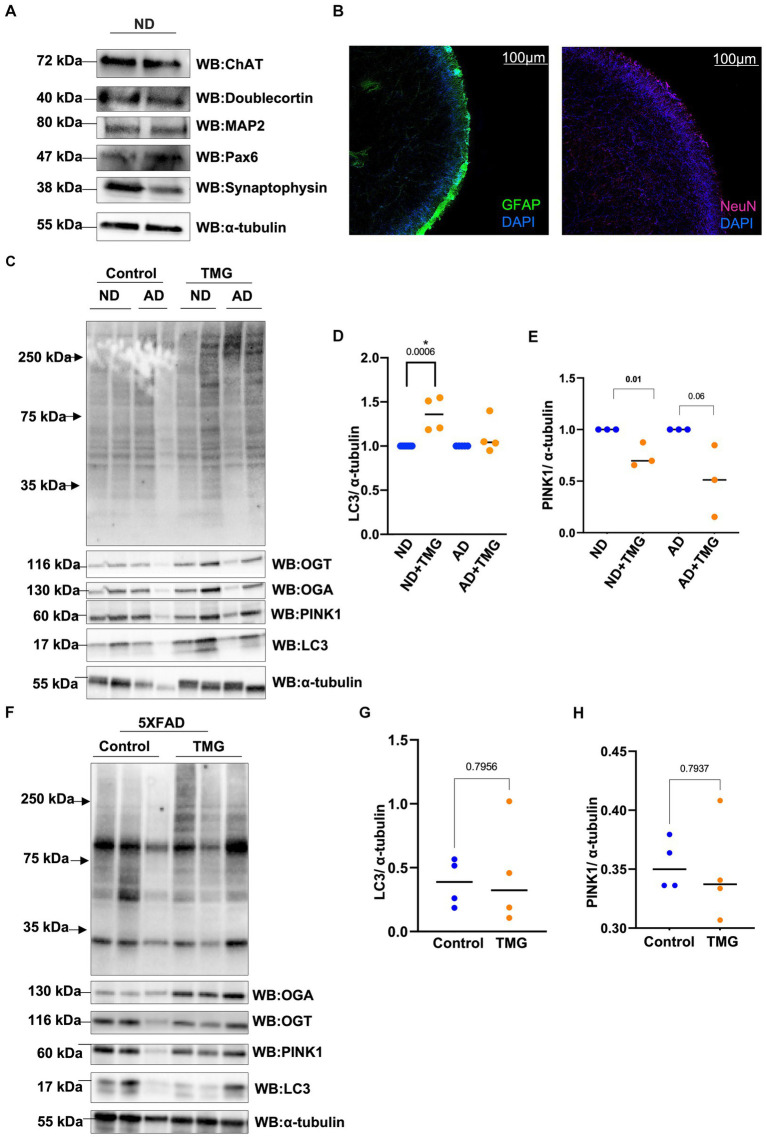
In AD, mitophagy response to O-GlcNAc alteration is altered. Representative Western blot analysis of differentiated organoids. Cholineacetyltransferase (ChAT), doublecortin, microtubule-associated protein 2 (MAP2), paired box protein (Pax-6), and synaptophysin **(A)**. Representative images from tissue clearing of ND cerebral organoid: Left image: glial fibrillary acidic protein (GFAP) in FITC and DAPI. Right image: neuronal nuclei (NeuN) stained with Cy5 and DAPI, 10X magnification **(B)**. Differentiated organoids from AD patients and healthy individuals of both genders were TMG treated for 2 weeks and lysed for Western blotting **(C)**. Densitometry plot of LC3 normalized to α-tubulin for organoids samples **(D)**. Densitometry plot of PINK1 normalized to α-tubulin for organoids samples **(E)**. Representative Western blot analysis of total brain lysate of 5XFAD mice subjected to intraperitoneal TMG injection for 1 month **(F)**. Densitometry plot of LC3 normalized to α-tubulin for 5XFAD mice samples **(G)**. Densitometry plot of PINK1 normalized to α-tubulin for 5XFAD mice samples **(H)**. ND, Non-demented; AD, Alzheimer’s disease. All experiments were performed with at least three biological replicates. Statistical significance was measured using unpaired *t*-test analysis, and the *p* value is indicated on the plots. ^*^Is added for *p* values that are significant (*p* < 0.05).

Because mitophagy is impaired in AD, we asked whether TMG elevates mitophagy proteins in a human *in vitro* AD organoid model. Organoid differentiation was validated via Western blot and immunostaining (Figures 9A,B). We treated differentiated brain organoids from sex- and age-matched AD patients and healthy individuals of both genders with TMG for 2 weeks. O-GlcNAc levels are significantly higher in the TMG-treated cells as expected (Figure 9C). LC3 is significantly elevated in TMG-treated organoids that are differentiated from normal individuals (Figures 9C,D). However, TMG failed to increase the expression of LC3 in organoids derived from Alzheimer’s patients. In the 5XFAD AD mouse model, there is no change in LC3 and PINK1 in 1-month TMG-treated organoids (Figures 9E-H).

## Discussion

Herein, we demonstrated the fundamental role of O-GlcNAcylation in effecting mitophagy. We also show that O-GlcNAcylation is responsive to mitochondrial stress. As previously reported, O-GlcNAcylation is elevated in response to many stressors, including heat shock (Kazemi et al., 2010) and oxidative stress (Groves et al., 2017), to promote cellular survival. In mitophagy, global cellular O-GlcNAcylation is decreased upon mitochondrial stress, while OGT protein level is decreased, and OGA is elevated. O-GlcNAc levels returned to the baseline after 8 h of mitochondrial stress. This O-GlcNAc change is an atypical response to stress; however, the O-GlcNAc response is elevated at the mitochondrial level. Mitochondrial O-GlcNAcylation, OGA, and OGT are elevated during mitochondrial stress. This indicates that organelle stress elevates O-GlcNAc compartmentally to respond to mitophagy stimulation. Furthermore, the elevation of both enzymes, OGT and OGA, suggests that either the import or the stability of the enzymes is elevated in mitochondria during mitochondrial stress. These data demonstrate that cellular response to mitochondrial stress modulates O-GlcNAc in a compartment-specific manner targeting the O-GlcNAc-processing enzymes to the stressed organelle. Potentially, changes in O-GlcNAc are promoting mitophagy or accelerating mitophagy steady state.

**Figure 10:**
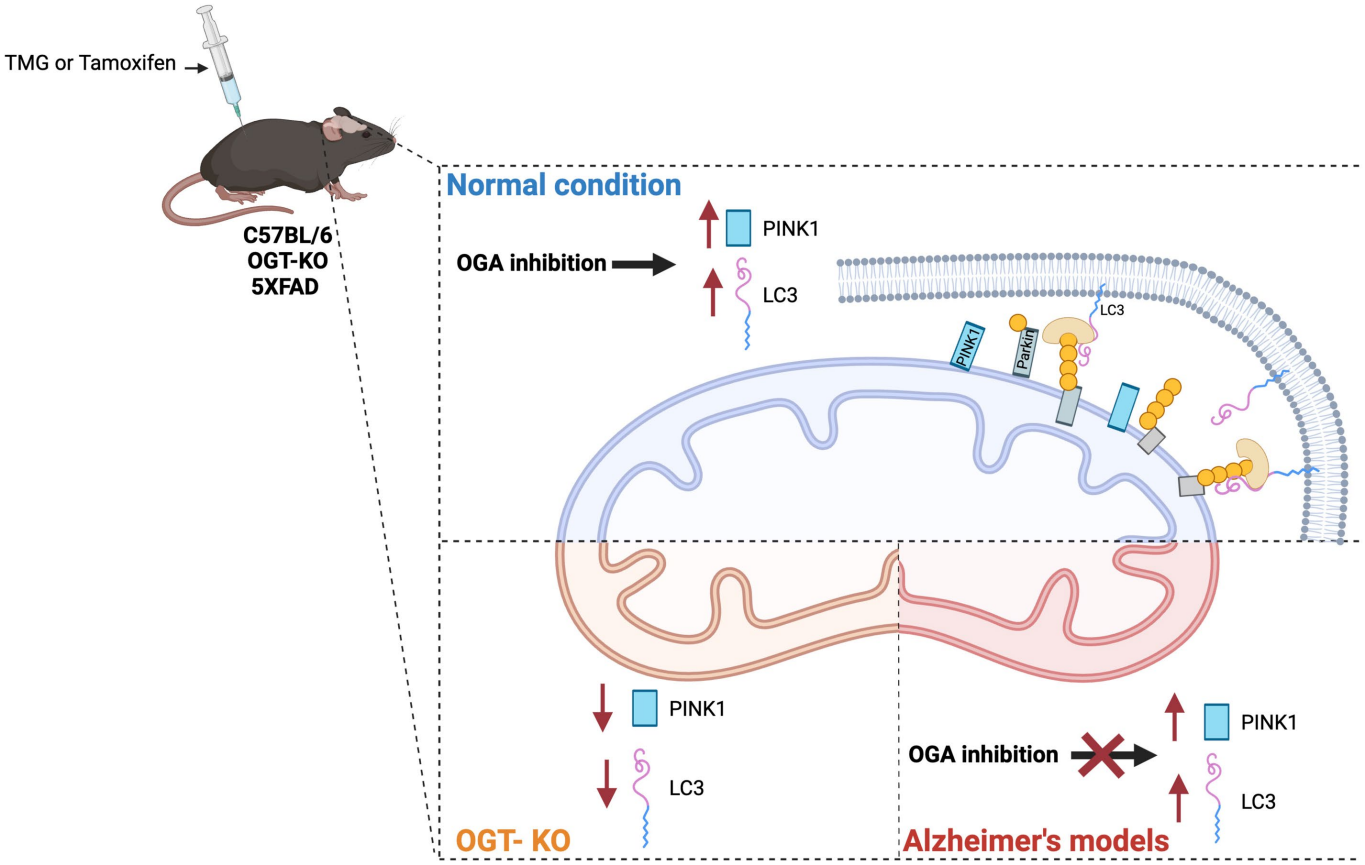
Effect of O-GlcNAc alterations on mitophagy. OGA inhibition stimulates PINK-dependent mitophagy in normal conditions via elevating LC3 and PINK1 at isolated mitochondria from SY5Y and normal mice brains treated with TMG. OGA inhibition has no effect on mitophagy in AD. OGT deficiency decreases mitophagy via reducing PINK1 and LC3 levels at mitochondria isolated from OGT-KO mice brains. OGT-KD decreased the protein expression of LC3 and PINK1 in SY5Y.

Impairment of mitochondrial proteostasis and exhaustion of mitochondrial retrograde signaling are implicated in the pathogenesis of AD (Kerr et al., 2017; Martín-Maestro et al., 2017; Sorrentino et al., 2017). In AD-affected brains and AD mouse models, PINK1 is downregulated (George et al., 2010; Wilhelmus et al., 2011; Du et al., 2017). Additionally, SH-SY5Y AD cybrid cells show impaired mitophagy due to PINK1 downregulation (Du et al., 2021). Restoring PINK1 levels rescues mitochondrial respiratory function, increases ATP levels, and attenuates oxidative stress in AD cybrid cells. We showed that TMG significantly elevates the concentration of PINK1 and LC3 levels in isolated mitochondria from SY5Y and mice brains indicating that TMG elevates mitophagosome formation (Figure 10). However, OGT-knockdown cells decrease PINK1 and LC3 indicating that low OGT expression abolished the formation of mitophagosome likely due to the reduction of PINK1 at the mitochondria and blocking mitophagy (Figure 10). The reduction in PINK1 in OGT-KO neurons is consistent with previous findings, showing that OGT-KO decreases PINK1 at the transcriptional level (Murakami et al., 2021). We demonstrated that PINK1 is modified by O-GlcNAc. However, site mapping PINK1 was challenging as PINK1 has low stability and is targeted by two degradation mechanisms, proteasome (Yamano and Youle, 2013), and mitophagy (Uoselis et al., 2023). Unfortunately, we were unable to enrich enough PINK1 to O-GlcNAc site-map via mass spectrometry even after blocking both degradation systems using proteasome inhibitor (MG-132) and lysosomal inhibitor (chloroquine) and OGA inhibitor TMG. Further research to uncover the mechanism of how O-GlcNAc regulates PINK1 function, stability, and its role in mitophagy is needed. Addressing the lack of mass spectrometry analysis on PINK1 is essential to provide in-depth mechanistic questions. Investigating whether O-GlcNAc regulates PINK1 interactions with mitophagy-related proteins like PARKIN and whether O-GlcNAcylation of PINK1 stabilizes it at the mitochondria is essential to provide a mechanistic explanation of how O-GlcNAc regulates mitophagy.

Activating mitochondria stress response provides neuroprotection and improves mitochondrial function (Zhang et al., 2020; Wang et al., 2021). Restoring mitophagy via the administration of urolithin A (UA) improves Alzheimer’s disease cognition, decreases AD-related toxic species aggregates, and restores the energetic status in AD neurons (Abisambra et al., 2013; D’Amico et al., 2021; Hou et al., 2024). Therefore, targeting mitophagy enhances the quality of mitochondria, making it a potential therapeutic target for AD. In fact, several synthetic chemicals and natural compounds are developed to target mitophagy eliminating dysfunctional mitochondria (Dyle et al., 2014; Tan et al., 2019). However, there are limited studies investigating the protective effect of OGA inhibitors in stimulating mitophagy. We showed that TMG significantly elevates LC3 protein levels in SY5Y cells and human brain organoids differentiated from normal individuals, while TMG failed to increase LC3 in organoids derived from AD patients. This could be due to the failure of OGA inhibition to stimulate the upstream transcriptional targets of mitophagy. Previously, we showed that the activity of ATF4, a master regulator of mitophagy-related genes such as PARKIN and LC3, is regulated by O-GlcNAc. The expression of ATF4 is elevated in TMG-treated SY5Y and organoids differentiated from normal individuals, while TMG has no impact on ATF4 expression in organoids derived from AD patients (Alghusen et al., 2023). These data indicate that OGA inhibition has a limited impact on mitochondrial quality control in AD compared to controls. Therefore, targeting OGA as a therapy fails to restore mitophagy in AD. Restoring mitophagy in AD improves mitochondrial function, energy production, and brain cognition in AD models, and OGA inhibition might not serve as a therapeutic target to restore mitophagy in AD.

## Data availability statement

The original contributions presented in the study are included in the article/Supplementary material, further inquiries can be directed to the corresponding author.

## Ethics statement

Ethical approval was not required for the studies on humans in accordance with the local legislation and institutional requirements because only commercially available established cell lines were used. The animal study was approved by University of Kansas Medical Center institutional animal care and use committee. The study was conducted in accordance with the local legislation and institutional requirements.

## Author contributions

IA: Conceptualization, Data curation, Investigation, Methodology, Visualization, Writing – original draft. MC: Investigation, Methodology, Writing – original draft. HW: Methodology, Writing – review & editing, Writing – original draft. TS: Investigation, Writing – review & editing. CG: Investigation, Writing – review & editing. HF: Investigation, Writing – review & editing, Writing – original draft. JS: Investigation, Writing – review & editing, Writing – original draft. SE: Investigation, Writing – review & editing, Writing – original draft. AD: Investigation, Writing – review & editing, Writing – original draft. XW: Investigation, Writing – review & editing. RS: Funding acquisition, Writing – review & editing, Writing – original draft. CS: Conceptualization, Funding acquisition, Project administration, Resources, Supervision, Writing – review & editing, Writing – original draft.
